# *Lycium barbarum* Polysaccharides Protect against Trimethyltin Chloride-Induced Apoptosis via Sonic Hedgehog and PI3K/Akt Signaling Pathways in Mouse Neuro-2a Cells

**DOI:** 10.1155/2016/9826726

**Published:** 2016-04-07

**Authors:** Wanyun Zhao, Xiaoqi Pan, Tao Li, Changchun Zhang, Nian Shi

**Affiliations:** Department of Health Toxicology, MOE Key Lab of Environment and Health, School of Public Health, Tongji Medical College, Huazhong University of Science and Technology, Wuhan 430030, China

## Abstract

Trimethyltin chloride (TMT) is a classic neurotoxicant that can cause severe neurodegenerative diseases. Some signaling pathways involving cell death play pivotal roles in the central nervous system. In this study, the role of Sonic Hedgehog (Shh) and PI3K/Akt pathways in TMT-induced apoptosis and protective effect of* Lycium barbarum* polysaccharides (LBP) on mouse neuro-2a (N2a) cells were investigated. Results showed that TMT treatment significantly enhanced apoptosis, upregulated proapoptotic Bax, downregulated antiapoptotic Bcl-2 expression, and increased caspase-3 activity in a dose-dependent manner in N2a cells. TMT induced oxidative stress in cells, performing reactive oxygen species (ROS) and malondialdehyde (MDA) excessive generation, and superoxide dismutase (SOD) activity reduction. TMT significantly decreased phosphorylated glycogen synthase kinase-3*β* (GSK-3*β*) and inhibited Shh and PI3K/Akt pathways. However, the addition of LBP upregulated GSK-3*β* phosphorylation, activated Shh and PI3K/Akt pathways, and eventually reduced apoptosis and oxidative stress caused by TMT. The interaction between Shh and PI3K/Akt pathways was clarified by specific PI3K inhibitor LY294002 or Shh inhibitor GDC-0449. Moreover, LY294002 and GDC-0449 pretreatment both induced phosphorylated GSK-3*β* downregulation and significantly promoted apoptosis induced by TMT. These results suggest that LBP could reduce TMT-induced N2a cells apoptosis by regulating GSK-3*β* phosphorylation, Shh, and PI3K/Akt signaling pathways.

## 1. Introduction

Trimethyltin chloride (TMT), an organotin compound and extremely neurotoxic agent, is generated in plastic and agricultural industries, producing polyvinyl chloride, silicone products, and fungicides [1]. Accumulating evidences suggested that TMT could lead to neuronal degeneration in humans and rodents [2–4]. TMT administration induces Parkinson's disease (PD) like symptoms (seizures), behavioral alterations (hyperactivity, tail mutilation, and aggression), and Alzheimer's disease (AD) like symptom (memory loss and learning impairment) [5]. Several studies indicated that TMT-induced neuronal degeneration is mainly due to cell apoptosis, activation of caspase family members, neuroinflammation, and oxidative stress [4, 6, 7]. Our early research has shown that there is cross talk and a balance between NF-*κ*B and MAPKs which may be involved in TMT-induced apoptosis on SH-SY5Y cells [8]. Despite numerous biochemical and neurochemical studies on TMT, its underlying neurotoxicity mechanism remains unknown.

Glycogen synthase kinase-3*β* (GSK-3*β*), GSK-3 isoform, is an important protein involved in signaling pathways, involving Notch, PI3K/Akt, Wnt, and Hedgehog. Overexpression of GSK-3*β* mRNA in neurons significantly promoted neuronal apoptosis [9]. Currently, GSK-3*β* is a new targeted treatment point in cancer [10]. Sonic Hedgehog (Shh), a mammalian member of the Hedgehog family, regulates the polarity of the central nervous system [11]. A recent study reported the interrelationship between Shh pathway and oxidative stress in vivo [12]. The activity of Shh signaling pathway in targeting gene expression is controlled by the Gli family (Gli1, Gli2, and Gli3), which is primarily regulated by GSK-3 [10]. PI3K/Akt signaling pathway serves a prosurvival function in neurons exposed to oxidative stress and apoptosis induced by various stimuli. The activated Akt confers cell survival by phosphorylating its cytoplasmic targets, such as GSK-3*β*, Bcl-2, Bax, and caspase-9 [13].

Fructus Lycii also called wolfberry is the dried mature fruit of* Lycium barbarum*. Fructus Lycii is a common traditional Chinese medicine used to protect the nervous system, improve immunity, regulate blood sugar, and protect the eyes [14, 15]. Fructus Lycii not only functions as an antioxidant agent but also exhibits other pleiotropic effects through its active component, namely,* Lycium barbarum* polysaccharides (LBP) [16, 17]. The polysaccharide exhibits neuroprotective effects on reducing cortical neuronal death in AD and is considered as a potential candidate for neurological disorders prevention [18]. Although several studies have reported the neuroprotective effect of LBP on neurodegenerative disease, the specific mechanism of the compound remains uncertain.

In this study, we evaluated whether TMT induced neuronal damage in N2a cells, in which GSK-3*β* expression and cross talk between Shh and PI3K/Akt pathways played crucial roles. Moreover, the beneficial effect of LBP on TMT-induced apoptosis in N2a cells was investigated.

## 2. Materials and Methods

### 2.1. Reagents and Instruments

TMT (>98% purity) was obtained from Tokyo Kasei Kogyo Co. (Tokyo, Japan). Dulbecco's modified Eagle's medium (DMEM) for cell culture was purchased from Hyclone. LBP (active ingredient > 31%) was provided by Shanghai Kangzhou Fungi Extract Co. (Shanghai, China). Antibodies against GSK-3*β*, p-GSK-3*β* (Ser9), Akt, p-Akt (Thr308), CyclinD1, C-Myc, GAPDH, and PI3K inhibitor LY294002 were acquired from Cell Signal Technology, Inc. (Beverly, MA). Antibodies against Gli1 and Shh were obtained from Abcam Inc. Antibodies against Bcl-2 and Bax were purchased from Santa Cruz Biotechnology Inc. (Santa Cruz, CA). Shh inhibitor GDC-0449 was obtained from Selleckchem, Inc. Reactive oxygen species (ROS) and caspase-3 activity assay kits were purchased from Beyotime Institute of Biotechnology (Shanghai, China). Malondialdehyde (MDA) and superoxide dismutase (SOD) kit were obtained from Nanjing Jiancheng Bioengineering Institute (Nanjing, China). PE-Annexin-V/7-AAD apoptosis detection kit was acquired from BD Pharmingen, Inc. (San Diego, CA). 3-(4,5-Dimethylthiazol-2-yl)-2,5-diphenyltetrazolium bromide (MTT) and Hoechst33258 dye were provided by Sigma Co.

### 2.2. Cell Culture and Treatment

Mouse Neuro-2a (N2a) cells were purchased from Shanghai Institute for Biological Sciences, Chinese Academy of Cell Resource Center (Shanghai, China). Cells were maintained at 37°C in a humidified atmosphere of 95% O_2_ and 5% CO_2_ in DMEM supplemented with 10% fetal bovine serum (FBS), penicillin (100 U/mL), and streptomycin (100 U/mL). The culture medium was replaced every 2 days. Cells were collected and seeded in new culture bottles. After incubation for 24 h, the medium was changed with serum-free medium containing different concentrations of TMT, LBP, and antagonists. TMT and LBP were dissolved in distilled water. The pharmacological inhibitors were dissolved in dimethyl sulfoxide (DMSO), and the final DMSO concentration in the medium was 0.1% to avoid any negative effects on cell survival. The cells were cotreated with LBP and TMT for 24 h or pretreated with LY294002 and GDC-0449 for 1 and 2 h, respectively, before TMT exposure for 24 h.

### 2.3. Cell Viability Assay

Cell viability was detected by MTT assays. Briefly, N2a cells were seeded in 96-well plates at a density of 10^4^ cells/well in DMEM (high glucose) containing 10% FBS. After seeding for 24 h, the medium was removed and replaced with 200 *μ*L of fresh medium containing serial concentrations of TMT (0.46875~120 *μ*M), LBP (300 *μ*g/mL), or antagonists. After 24 h of incubation, the cells were treated with 20 *μ*L of fresh medium containing 0.5 mg/mL MTT and incubated at 37°C for 4 h. The solution was removed, and about 150 *μ*L of DMSO was added to each well to dissolve the purple formazan crystals. Absorbance was determined at 490 nm by using a microplate reader. Results were expressed as the percentage of MTT reduction relative to the absorbance of control cells.

### 2.4. Flow Cytometric Analysis

Early stages of apoptosis were analyzed using a PE-Annexin-V/7-AAD apoptosis detection kit. After exposure to the test agents for 24 h, cells were harvested, washed with PBS and binding buffer, and stained for 10 min with PE and 7-AAD (dissolved in the binding buffer provided by the manufacturer). The numbers of PE-positive/7-AAD-negative, PE-positive/7-AAD-positive, and PE-negative/7-AAD-positive cells and unlabeled cells were determined with a Becton-Dickinson FACS flow cytometer. Data were analyzed using Cell Quest software, and 10,000 events were assessed for each independent experiment. The numbers of 7-AAD-positive/PE-positive (necrotic cells, quadrant I), 7-AAD-positive/PE-negative (late apoptotic, quadrant II), AV-negative/PI-positive (early apoptotic, quadrant IV), and unlabeled (intact cells, quadrant III) cells were determined.

### 2.5. Immunocytofluorescence Staining Analysis

Cells were incubated on pretreated coverslips. After exposure to TMT (1.875, 3.75, and 7.5 *μ*M) and/or LBP (300 *μ*g/mL) for 24 h, the cells were fixed with 4% paraformaldehyde for 20 min at room temperature. The slides were incubated for 20 min in 0.3% Triton X-100 diluted with 0.1 M PBS for permeabilization, rinsed three times in PBS, and blocked with normal goat serum for 20 min at room temperature. Rabbit anti-mouse p-Akt and p-GSK-3*β* polyclonal antibodies (1 : 300 and 1 : 500) were used as primary antibodies. The slides were then incubated with primary antibodies at 4°C overnight and reacted with FITC-conjugated goat anti-rabbit IgG antibody (1 : 100) for 1 h at room temperature. DAPI dye was used for nuclear staining before washing, and the coverslips were covered on the slides with an antifade mounting medium. Images were captured and digitized under an inverted fluorescence microscope (IX71, Olympus, Japan).

### 2.6. DNA Condensation and Nuclear Fragmentation

Hoechst33258 fluorescent dye was used to determine DNA condensation and nuclear fragmentation of apoptotic cells. N2a cells were seeded in a six-well plate at a density of 5 × 10^4^ cells/well. After treatment with or without TMT and LBP for 24 h at 37°C, the cells were washed with PBS and fixed with 4% paraformaldehyde for 20 min at 4°C. The fixed cells were washed with PBS and stained with 5 *μ*g/mL Hoechst33258 for 10 min. After incubation, the cells were washed again with PBS. Images were obtained under an inverted fluorescence microscope (IX71, Olympus, Japan).

### 2.7. Detection of Intracellular ROS Accumulation

A fluorescence probe (DCFH-DA) was used to determine ROS in N2a cells. DCFH-DA was deacetylated in cells, where it could react with free radicals (H_2_O_2_) and be converted into the fluorescent product DCF. N2a cells were incubated with 5 × 10^4^ cells/well in a six-well plate, at 37°C with or without TMT for 24 h to determine intracellular ROS level. The probe was added to the culture medium and washed three times with PBS. Images were obtained after 30 min of incubation (IX71, Olympus, Japan). Fluorescence intensity was also detected. N2a cells (10^4^ cells/well in a 96-well plate) were incubated with different concentrations of TMT for 24 h. After loading the probes for 30 min, intoxicated cells were washed three times with PBS. Fluorescence intensity was examined at Ex = 488 nm and Em = 525 nm by using a microplate reader.

### 2.8. Lipid Peroxidation Measurement

MDA content was determined by double heating method, which is based on colorimetric determination of the purple color generated by the reaction of thiobarbituric acid (TBA) with MDA. The collected cells were mixed with TBA (10% w/v) solution and then boiled for 15 min. The samples were centrifuged at 3000 rpm for 10 min and the supernatant was transferred to a new tube and reacted with the TBA (0.67% w/v) solution. After boiling for 15 min, the samples were cooled to room temperature and absorbance was determined at 532 nm with a microplate reader.

### 2.9. Antioxidant Determination

SOD activity was measured based on the inhibition extent of amino blue tetrazolium formazans in the mixture of nicotinamide adenine dinucleotide, phenazine methosulphate, and nitroblue tetrazolium. The color intensity of chromogen was determined at 560 nm. Activity unit was defined as the number of enzymes that inhibited 50% of NBT reduction per milligram of protein.

### 2.10. Western Blot Analysis

Cells were lysed in RIPA lysis buffer containing 1% PMSF. Total proteins were obtained through centrifugation. After quantification of protein concentration by using the BCA method, the total proteins were electrophoresed in 12% SDS-PAGE gels and transferred onto nitrocellulose filter membranes. The membranes were blocked with 5% BSA for 1.5 h, and the blots were incubated with primary antibodies against GSK-3*β*, p-GSK-3*β*, Akt, p-Akt, CyclinD1, C-Myc, Gli1, Shh, Bcl-2, Bax, and GAPDH (1 : 1000) at 4°C overnight. The membranes were washed three times with TBST for 5 min each, incubated with goat anti-rabbit (1 : 2500) IgG labeled with horseradish peroxidase for 1 h, and then washed. Immunoreactive proteins were detected using the ECL detection system, and densitometric analysis of immunoblots was performed with the Gel pro 3.0 software. GAPDH was used as the protein loading control.

### 2.11. Caspase-3 Activity Assay

Caspase-3 activity was measured through the cleavage of the chromogenic caspase substrate, namely, acetyl-Asp-Glu-Val-Asp p-nitroanilide (Ac-DEVD-pNA). Approximately 50 *μ*g of the total protein was added into a reaction buffer containing Ac-DEVD-pNA (2 mM) and incubated for 2 h at 37°C. Absorbance of the yellow pNA cleaved from the corresponding precursor was spectrometrically determined at 405 nm. Specific caspase activity was normalized to the total protein concentration of cell lysates and then expressed as fold increase over baseline caspase activity, which was determined from control cells cultured in DMEM with 10% FBS or vehicle treated control cells.

### 2.12. Statistical Analysis

All results were expressed as mean ± SD. All experiments were performed independently at least four times. Statistical analysis was conducted with SPSS18.0 software. Means were compared by one-way analysis of variance, and results between groups were compared using the Bonferroni test. Gait score was analyzed using Mann-Whitney *U* test. Significance was defined at *P* < 0.05.

## 3. Results

### 3.1. Protective Effect of LBP on Cytotoxicity Induced by TMT in N2a Cells

**Figure 1:**
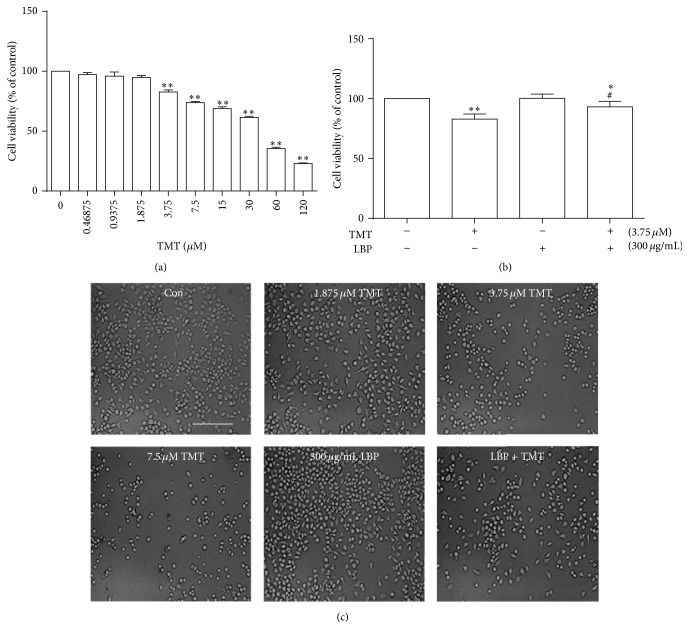
Cytotoxicity of TMT on N2a cells. N2a cells were treated with different concentrations of TMT (0.46875~120 *μ*M) for 24 h. Cell viability was detected by MTT assay as described in Section 2 (a). Cells were treated with or without 300 *μ*g/mL LBP and 3.75 *μ*M TMT for 24 h, and cell viability was also determined by MTT assay (b). The morphological changes in N2a cells after TMT and/or LBP treatment for 24 h were observed under an inverted light microscope, scale bar: 50 *μ*m (c). Cell survival rate was calculated and represented as mean ± SD (*n* = 6). ^*∗*^ *P* < 0.05, ^*∗∗*^ *P* < 0.01 compared with the control group; ^#^ *P* < 0.05 compared with the TMT group.

Cytotoxicity of TMT on N2a cells was determined by MTT method. As shown in Figure 1(a), exposure to different concentrations of TMT (0.46875~120 *μ*M) for 24 h dose-dependently reduced cell viability. By contrast, 300 *μ*g/mL LBP significantly enhanced cell viability induced by treatment with 3.75 *μ*M TMT (Figures 1(a) and 1(b)). The cytotoxicity of TMT and the effect of LBP were also confirmed through morphological observation (Figure 1(c)). Under an inverted light microscope, the cells were retracted and rounded after exposure to different concentrations of TMT (1.875, 3.75, and 7.5 *μ*M) for 24 h. Morphological changes were observed in N2a cells treated with 300 *μ*g/mL LBP and 3.75 *μ*M TMT. These results indicated that cell viability was inhibited by TMT treatment, and LBP played a protective role in TMT-induced cell death.

### 3.2. LBP Attenuates N2a Cells Apoptosis Caused by TMT

**Figure 2:**
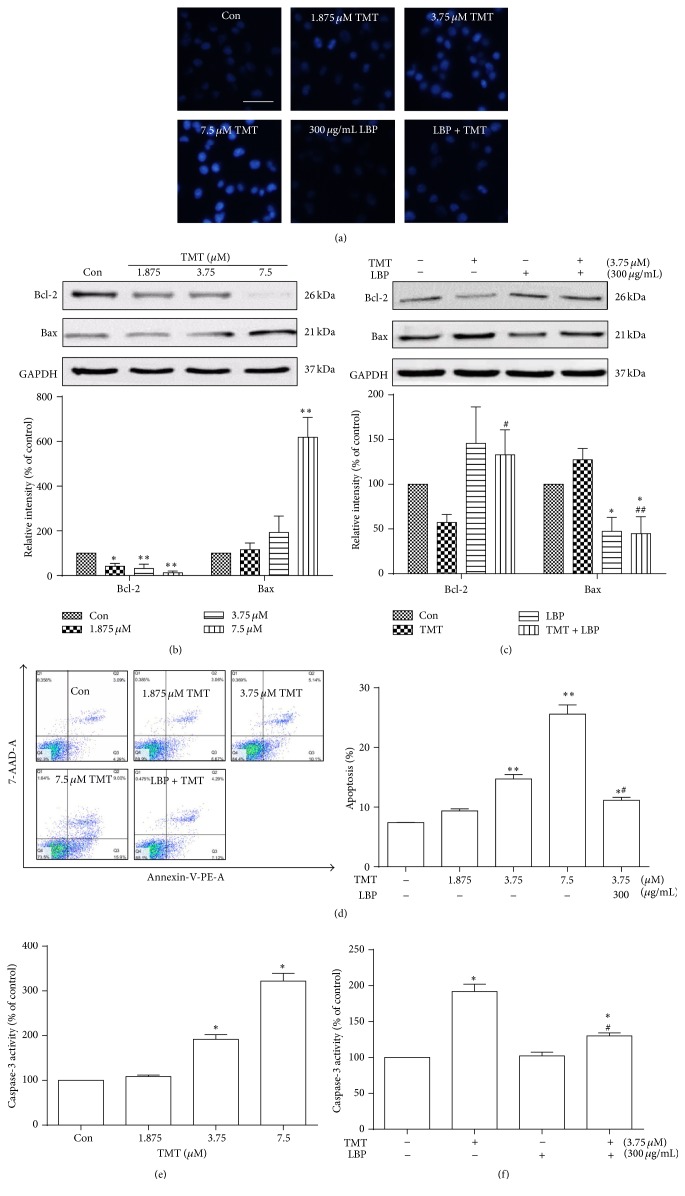
Protective effects of LBP on TMT-induced apoptosis in N2a cells. N2a cells were treated with different concentrations of TMT (1.875, 3.75, and 7.5 *μ*M) or cotreated with LBP (300 *μ*g/mL) for 24 h. DNA condensation and fragmentation in N2a cells were observed using Hoechst33258 staining under an inverted fluorescent microscope, scale bar: 25 *μ*m (a). Effects of TMT treatment on expression of Bcl-2 and Bax as determined through Western blot analysis. Total protein extracts were prepared after incubating the cells with 1.875~7.5 *μ*M TMT for 24 h (b). Effects of LBP on the expression of Bcl-2 and Bax induced by TMT, as determined by Western blot analysis. Total protein extracts were prepared after incubating the cells with 300 *μ*g/mL LBP and 3.75 *μ*M TMT for 24 h (c). All Western blot analysis results were expressed as fold changes of optical density, with GAPDH as the internal control. The mean protein expression of control is designated as 1 in the graph. After incubation with 1.875~7.5 *μ*M TMT for 24 h, cells were collected and stained with PE-Annexin-V and 7-AAD for 5 min. Samples were analyzed by flow cytometry. The proportions of living and dead cells were determined by flow cytometric analysis of PE-Annexin-V and 7-AAD-labeled cells. Live cells are unlabeled with PE and 7-AAD (Q3), whereas PE labeling (Q4) represents the population of early apoptosis. Cells showing PE and 7-AAD double labeling (Q2) represent those that have already died due to apoptosis. Ten thousand cells were analyzed in each sample. The percentages of the total apoptosis analyzed were determined (d). The effects of TMT alone (e) or TMT with LBP intervention (f) on caspase-3 activity were assayed by ELISA method. Each bar represents mean ± SD (*n* ≥ 3). ^*∗*^ *P* < 0.05, ^*∗∗*^ *P* < 0.01 compared with the control group; ^#^ *P* < 0.05, ^##^ *P* < 0.01 compared with the TMT-treated group.

DNA damage induced by different concentrations of TMT (1.875, 3.75, and 7.5 *μ*M) and LBP intervention were determined by Hoechst33258 staining in N2a cells (Figure 2(a)). DNA condensation and fragmentation occurred in N2a cells treated with 3.75 and 7.5 *μ*M TMT for 24 h. Meanwhile, these apoptosis features and TMT-induced morphological alterations were inhibited by LBP at a concentration of 300 *μ*g/mL. Western blot analysis showed that incubation with 7.5 *μ*M TMT for 24 h significantly decreased Bcl-2 protein expression and increased Bax protein level (Figures 2(b) and 2(c)). However, in the cells cotreated with 300 *μ*g/mL LBP and 3.75 *μ*M TMT, Bcl-2 expression was upregulated, and Bax expression was downregulated (*P* < 0.01). Flow cytometry using FITC-conjugated PE and 7-AAD double staining assay was further employed to quantitatively determine apoptosis induced by TMT and the protective effect of LBP. As shown in Figure 2(d), TMT markedly enhanced apoptosis in N2a cells, compared with the control (*P* < 0.01) and LBP reduced the TMT-induced apoptosis (*P* < 0.05). The activity of caspase-3 significantly increased after TMT treatment for 24 h, whereas LBP downregulated caspase-3 activity (*P* < 0.05). All results indicated TMT induced cell apoptosis in a concentration-dependent manner. Moreover, LBP effectively attenuated TMT-induced apoptosis (Figures 2(e) and 2(f)).

### 3.3. LBP Suppresses Oxidative Damage Induced by TMT in N2a Cells

**Figure 3:**
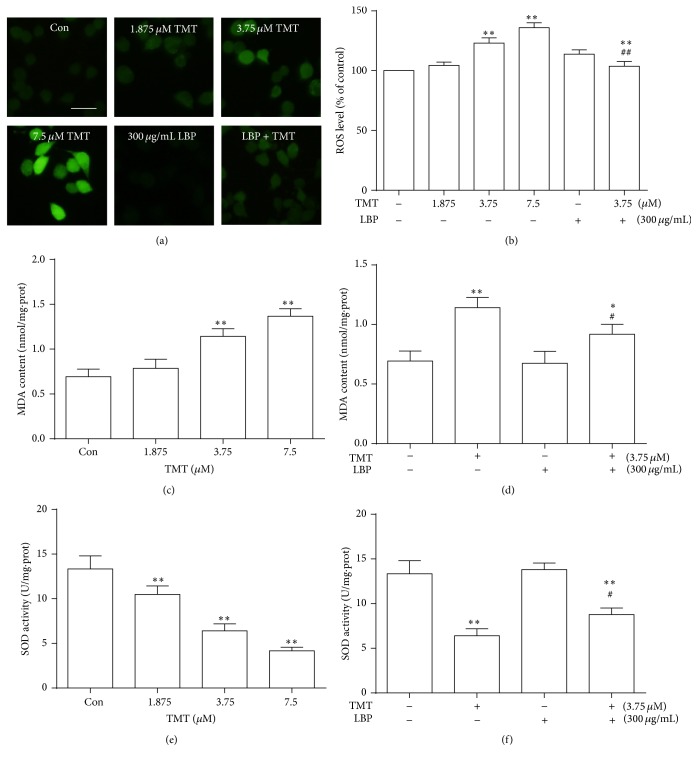
Effects of LBP on ROS, MDA generation, and SOD activity induced by TMT in N2a cells. Intracellular ROS was evaluated by DCFH-DA detection. Fluorescent photographs are shown after exposure to TMT (1.875, 3.75, and 7.5 *μ*M) and 3.75 *μ*M TMT with or without LBP (300 *μ*g/mL) cotreatment for 24 h, scale bar: 25 *μ*m (a). Furthermore, ROS, MDA, and SOD productions induced by TMT treatment (0, 1.875, 3.75, and 7.5 *μ*M) alone for 24 h or 3.75 *μ*M TMT with LBP (300 *μ*g/mL) were quantified (b–f). The results are expressed as mean ± SD (*n* = 6). ^*∗*^ *P* < 0.05, ^*∗∗*^ *P* < 0.01 compared with the control group; ^#^ *P* < 0.05, ^##^ *P* < 0.01 compared with the TMT-treated group.

In order to observe the effects of LBP on oxidative stress induced by different concentrations of TMT (1.875, 3.75, and 7.5 *μ*M) for 24 h, ROS level was examined in N2a cells through fluorescent dye DCFH-DA. As shown in Figure 3(b), ROS in the groups treated with 3.75 and 7.5 *μ*M TMT significantly increased compared with those in the control group (*P* < 0.01). Similar results were observed in immunofluorescence staining analysis (Figure 3(a)). After TMT treatment, MDA level significantly increased and SOD content decreased. These two targets showed a concentration-dependent trend in N2a cells (Figures 3(c) and 3(e)). However, when culture cells were cotreated with LBP and TMT for 24 h, ROS and MDA levels significantly decreased (Figures 3(a), 3(b), and 3(d)), whereas SOD activity had a significant increase (Figure 3(f)). These results suggested that LBP exhibited a protective role in TMT-induced oxidative damage in N2a cells.

### 3.4. Inhibition of Shh and PI3K/Akt Signaling Pathways by TMT in N2a Cells

**Figure 4:**
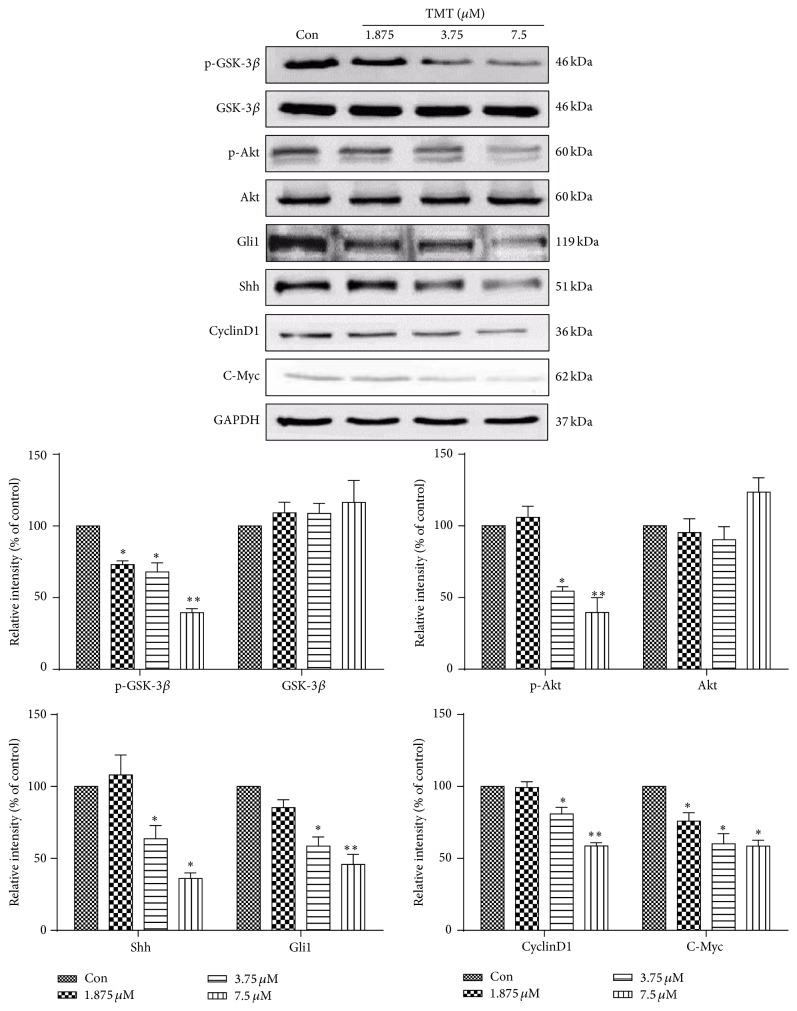
TMT-induced Shh and PI3K/Akt inhibition. N2a cells were incubated with 1.875, 3.75, and 7.5 *μ*M TMT for 24 h. Different protein expressions levels were determined through Western blot analysis. The results are expressed as fold increases of optical density with the internal control. The mean protein expression of control group is designated as one in the graph. Each bar represents mean ± SD (*n* = 3). ^*∗*^ *P* < 0.05, ^*∗∗*^ *P* < 0.01 compared with the control group.

To determine whether the Shh and PI3K/Akt signaling pathways regulate TMT-induced apoptosis, we determined the levels of Shh, Gli1, and p-Akt through Western blot analysis. Meanwhile, p-GSK-3*β*, CyclinD1, and C-Myc were evaluated to determine the downstream effects of signaling pathways through Western blot analysis. The cells were incubated with different concentrations of TMT (1.875, 3.75, and 7.5 *μ*M) for 24 h. As shown in Figure 4, the levels of Shh, Gli1, and p-Akt significantly decreased (*P* < 0.05), whereas those of p-GSK-3*β*, CyclinD1, and C-Myc, which are downstream proteins of Shh and PI3K/Akt signaling pathways, significantly decreased. Therefore, TMT treatment concentration-dependently inhibited the Shh and PI3K/Akt signaling pathways.

### 3.5. Effect of LBP on TMT-Induced Inhibition of Shh and PI3K/Akt Signaling in N2a Cells

**Figure 5:**
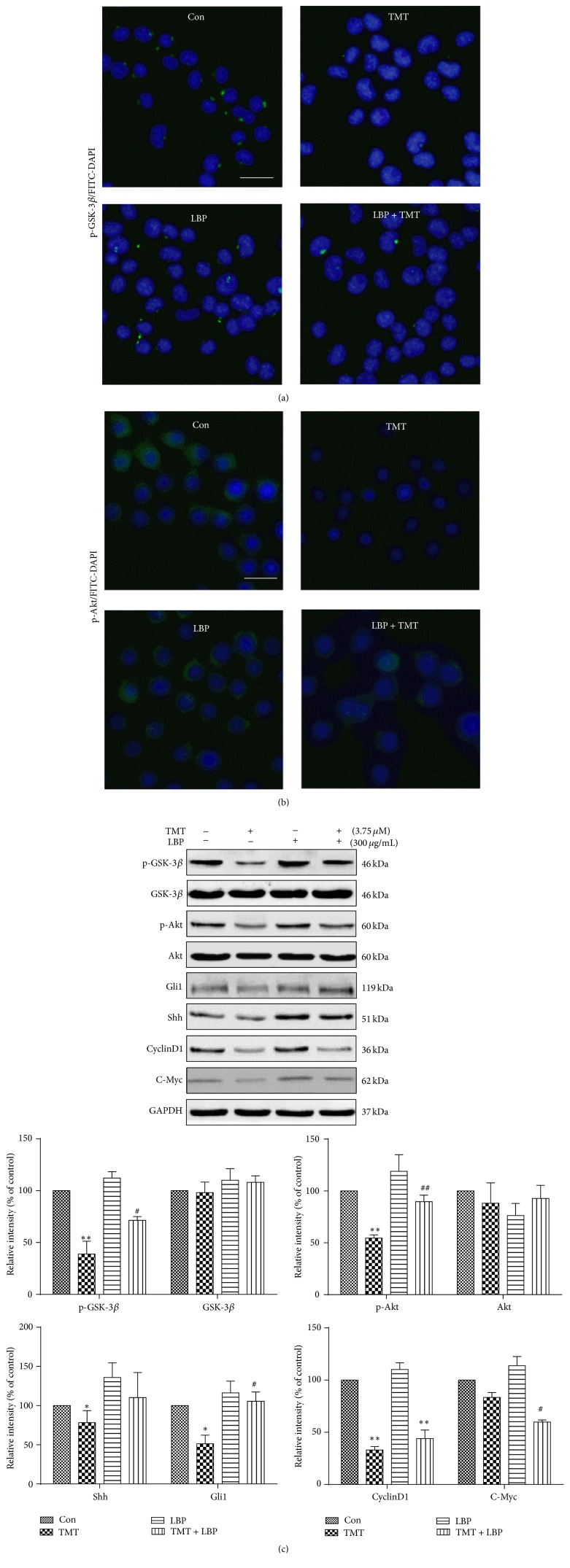
Effect of LBP on TMT-induced signaling inhibition. The cells were incubated with 300 *μ*g/mL LBP and 3.75 *μ*M TMT for 24 h. Fluorescent photographs are shown after exposure to TMT with or without LBP for 24 h, scale bar: 25 *μ*m (a and b). Different protein levels expressions were determined through Western blot analysis. The results are expressed as fold increases of optical density with the internal control. The mean protein expression of control group is designated as one in the graph (c). Each bar represents mean ± SD (*n* = 3). ^*∗*^ *P* < 0.05, ^*∗∗*^ *P* < 0.01 compared with the control group; ^#^ *P* < 0.05, ^##^ *P* < 0.01 compared with the TMT-treated group.

N2a cells were cotreated with 3.75 *μ*M TMT and 300 *μ*g/mL LBP for 24 h to clarify the effects of LBP on TMT-induced signaling inhibition. As shown in Figure 5(c), Western blot analysis results indicated that phosphorylated GSK-3*β*, phosphorylated Akt, and Gli1 protein expression significantly increased, and C-Myc protein level significantly decreased in TMT and LBP treatment, compared with that in TMT alone treatment. GSK-3*β* was also partly inhibited in treatment with LBP (*P* < 0.05). These findings were confirmed through immunofluorescence staining analysis (Figures 5(a) and 5(b)). All the results demonstrated the protective effect of LBP in TMT-induced Shh and PI3K/Akt signaling inhibition.

### 3.6. Roles of Shh and PI3K/Akt Signaling Pathways in TMT-Induced Apoptosis in N2a Cells

**Figure 6:**
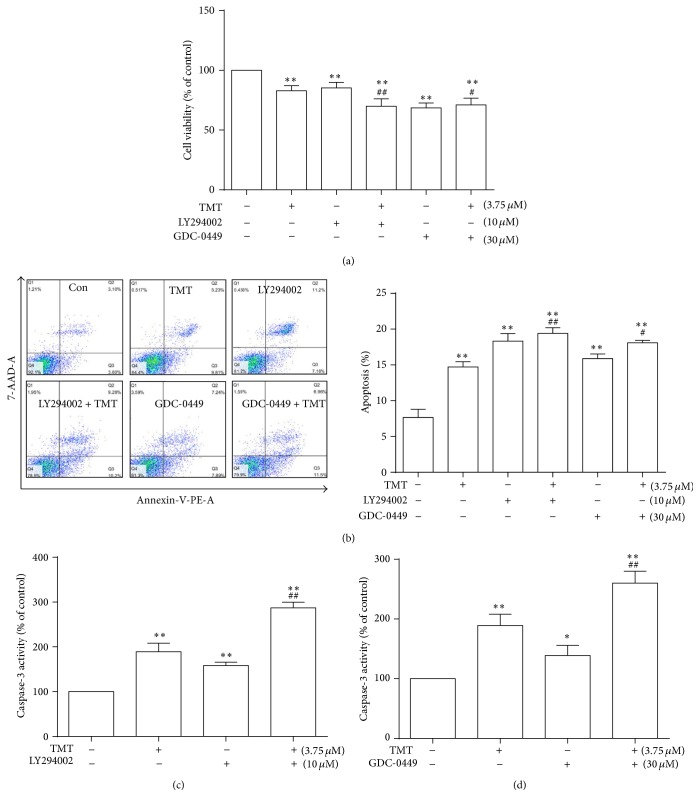
Effects of antagonists on TMT-induced apoptosis in N2a cells. N2a cells were pretreated with or without different inhibitors at different time points in the following concentrations: LY2940002, 10 *μ*M; GDC-0449, 30 *μ*M, followed by incubation with or without 3.75 *μ*M TMT for another 24 h. Cell survival rate was calculated and represented as mean ± SD (*n* = 6) (a). Cells were collected and stained with PE-Annexin-V and 7-AAD for 5 min. The samples were analyzed through flow cytometry. The percentage of apoptosis cells based on the total cell population analyzed was determined (b). Effects of inhibitors on TMT-treated N2a cells; caspase-3 activity was determined through ELISA (c and d). Each bar represents mean ± SD (*n* ≥ 3). ^*∗*^ *P* < 0.05, ^*∗∗*^ *P* < 0.01 compared with the control group; ^#^ *P* < 0.05, ^##^ *P* < 0.01 compared with the TMT-treated group.

Shh and PI3K/Akt signaling pathways were inhibited using LY294002 and GDC-0449 to determine the effect of their inhibition on apoptosis induction. LY294002 acts as a highly selective inhibitor of PI3K in vivo, and GDC-0449 targets the Hedgehog signaling pathway by blocking the activities of Hedgehog-ligand cell surface receptors and suppressing Hedgehog signaling. Pretreatment with LY294002 (1 h, 10 *μ*M) and GDC-0449 (2 h, 30 *μ*M) promoted cytotoxicity induced by 3.75 *μ*M TMT (Figure 6(a)). Meanwhile, flow cytometry results showed that pretreatment with LY294002 and GDC-0449 significantly increased TMT-induced apoptosis (Figure 6(b)). Caspase-3 activity significantly increased in 3.75 *μ*M TMT exposure groups pretreated with inhibitors, compared with that in control group or TMT alone treatment group (Figures 6(c) and 6(d)). Hence, pretreatment with both inhibitors caused a statistical increase in apoptosis induced by TMT.

### 3.7. Effects of Shh and PI3K/Akt Signaling Pathways on Oxidative Stress Induced by TMT in N2a Cells

**Figure 7:**
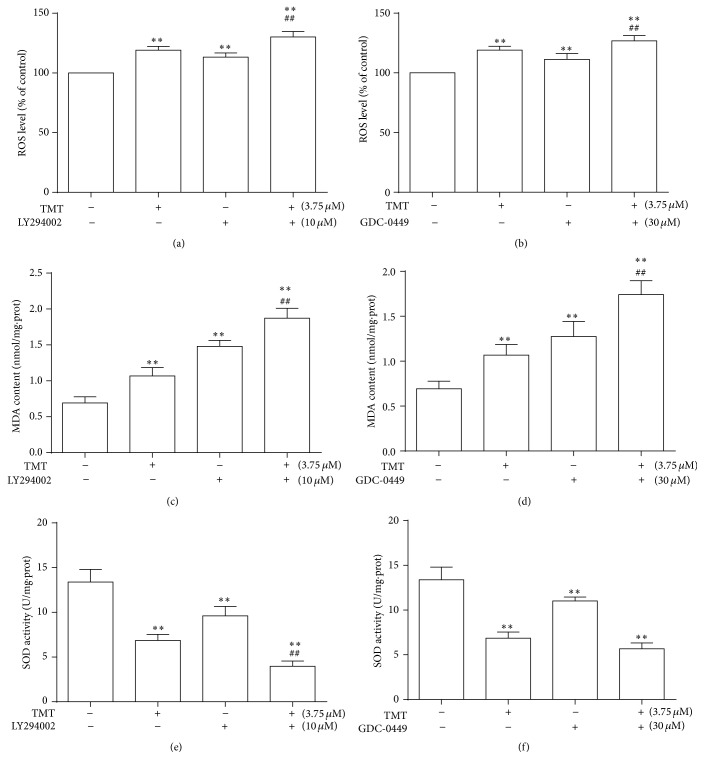
Effects of antagonists on ROS, MDA generation, and SOD activity caused by TMT in N2a cells. Cells were cultured with 3.75 *μ*M TMT, pretreated with or without different inhibitors at different time points in different concentration (LY2940002, 10 *μ*M; GDC-0449, 30 *μ*M), and quantified to evaluate ROS, MDA, and SOD productions. Results are expressed as mean ± SD (*n* = 6). ^*∗*^ *P* < 0.05, ^*∗∗*^ *P* < 0.01 compared with the control group; ^#^ *P* < 0.05, ^##^ *P* < 0.01 compared with the TMT-treated group.

To confirm the role of Shh and PI3K/Akt signaling pathways on oxidative stress induced by 3.75 *μ*M TMT for 24 h, we quantified the levels of ROS, MDA, and SOD. Figures 7(a) and 7(b) indicated that ROS level in groups, pretreated with LY294002 (1 h, 10 *μ*M) and GDC-0449 (2 h, 30 *μ*M) inhibitors, significantly decreased compared with that in control group or TMT-treated group (*P* < 0.01). Consistently, MDA level significantly increased and SOD content decreased in N2a cells treated with inhibitors (Figures 7(c), 7(d), 7(e), and 7(f)). These data are consistent with the notion that inhibition of Shh and PI3K/Akt signaling pathways could activate TMT-induced oxidative damage.

### 3.8. Cross Talk between Shh and PI3K/Akt Signaling Pathways

**Figure 8:**
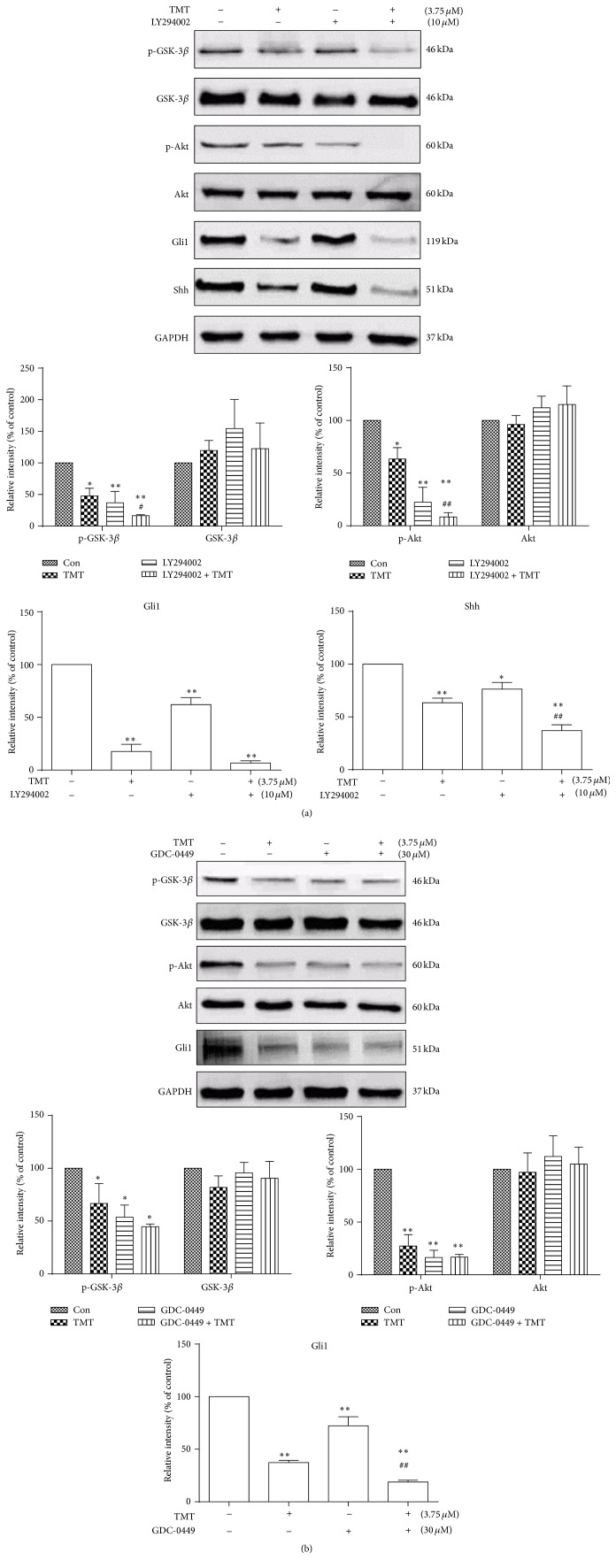
Effects of different antagonists on Shh and PI3K/Akt signaling pathways. Cells were pretreated with or without different inhibitors at different time points in the following concentrations: LY2940002, 10 *μ*M; GDC-0449, 30 *μ*M, before TMT treatment. After incubation with 3.75 *μ*M TMT for another 24 h, the expression levels of proteins were determined through Western blot analysis. The mean protein expression of control group is designated as 1 in the graph. Each bar represents mean ± SD (*n* = 3). ^*∗*^ *P* < 0.05, ^*∗∗*^ *P* < 0.01 compared with the control group; ^#^ *P* < 0.05, ^##^ *P* < 0.01 compared with the TMT-treated group.

To demonstrate the interaction between Shh and PI3K/Akt pathways, we used specific inhibitors and evaluated possible alteration of each pathway. N2a cells were incubated with inhibitors of Shh (GDC-0449) or PI3K/Akt (LY294002) for 2 h before 3.75 *μ*M TMT treatment for 24 h. As shown in Figure 8, the inhibition of the PI3K/Akt signaling pathway with LY294002 significantly reduced Shh and GSK-3*β* protein expression compared with TMT treatment (*P* < 0.05). Meanwhile, GDC-0449 pretreatment significantly inhibited Gli1 protein expression compared with TMT alone exposure (*P* < 0.01) and had no significant effect on p-GSK-3*β* and p-Akt protein expression. These results indicated that the inhibition of PI3K/Akt pathway could affect GSK-3*β* phosphorylation and the activation of Shh pathway.

## 4. Discussion

The study was focused on the signaling pathways that contributed to TMT-induced cell apoptosis. In particular, the protective effect of LBP and the roles of Shh and PI3K/Akt signaling pathways in TMT-induced N2a cells apoptosis were studied. Neuro-2a cell lines which derive from mouse neuroblastoma can express neuronal phenotype and are a common model for studies on the mechanism of nervous systemic disease [19].

TMT exposure exhibited evident cytotoxicity and decreased cell viability in N2a cells in a dose-dependent manner. Thus, the doses of 1.875, 3.75, and 7.5 *μ*M were selected as TMT exposure concentrations in our study, and relatively similar concentrations were applied in our previous experiments [8]. But there was some slight difference in concentration choice, which may be due to different cytotoxicity of TMT on various species cell lines. Except for this, TMT was proved to induce apoptosis in N2a cells by significantly upregulating Bax expression, downregulating Bcl-2 expression, and increasing caspase-3 activity. Oxidative stress is closely related not only to neurodegenerative disorders, but also to apoptosis [4, 20, 21]. In the study, TMT induced ROS generation and lipid peroxidation in N2a cells. Thus, we infer that TMT regulates apoptotic related genes, activates caspase cascade, causes oxidative damage, and finally induces cell apoptosis.

PI3K/Akt signaling is a membrane receptor signaling pathway related to intracellular transduction. Previous study showed that PI3K/Akt pathway could be involved in TMT-induced neuronal damage or apoptosis [22]. The pathway not only regulates various signaling molecules, but also is regulated by signaling molecules, particularly regulatory molecules, such as ROS [23, 24]. Hence, ROS overproduction induced by TMT in N2a cells could be considered as a stimulus of PI3K/Akt pathway activation. Although some studies showed that TMT had a completely opposite effect on PI3K/Akt pathway [22, 25], TMT treatment in N2a cells inhibited Akt phosphorylation in the study. In addition, the inhibition of PI3K/Akt pathway by LY294002 increased ROS and MDA generations and decreased SOD levels, which enhanced oxidative damage and promoted cell apoptosis in N2a cells. Thus, it was confirmed that PI3K/Akt signaling pathway is a protective regulator in response to TMT exposure in N2a cells. TMT neurotoxicity on N2a cells may be related to blocking the protective signaling pathway PI3K/Akt.

Sonic Hedgehog (Shh) pathway plays a vital role in brain development, neural stem cell proliferation, and neuronal regeneration and repair [26, 27]. The activity of the Shh signaling pathway targeting genes expression is controlled by the Gli family (Gli1, Gli2, and Gli3). The multiplication and proliferation of effector molecules (such as CyclinD1 and C-Myc) in tumor cells are proved to be the target genes or downstream molecules of Hedgehog signaling pathway [28]. There are studies showing that the activation of Shh signaling protects cortical neurons against oxidative stress, suggesting a potential role of Shh for clinic treatments of brain ischemia and neurodegenerative disorders [12]. Hence, Shh signaling change in our model was mainly studied. In our study, Shh pathway was suppressed by TMT treatment, confirming that Gli1, CyclinD1, and C-Myc expression significantly decreased in N2a cells. The suppression of Shh pathway reduced cell viability and enhanced oxidative damage and apoptosis, which were similar to PI3K/Akt inhibition. Thus, Shh pathway also had a protective effect on TMT-induced neurotoxicity in N2a cells, just like PI3K/Akt. We therefore infer that the reasons why TMT inhibited Shh expression may be excessive ROS generation, lipid peroxidation, and TMT-induced PI3K/Akt signaling pathway blockage, which destroys the natural repair system of body.

GSK-3*β* is not only a downstream effector kinase of PI3K/Akt pathway, but also an important kinase of Shh signaling pathway [29]. GSK-3*β* is suggested as one of the key regulators of the Gli family [10]. We drew close attention to investigate the relationship between Shh and PI3K/Akt pathways by using each of the inhibitors GDC-0449 and LY294002 in TMT-treated N2a cells. Results suggested that TMT inhibited GSK-3*β* phosphorylation which activated GSK-3*β* signaling. The distribution of p-GSK-3*β* was throughout the cytoplasm, whereas p-Akt was distributed in cytoplasm with point shape. Shh pathway was inhibited when PI3K/Akt pathway was blocked. Similarly, Akt phosphorylation was decreased when Shh pathway was inhibited. The similar findings in other studies showed that Shh acted on PI3K/Akt, and Akt had a regulatory role in the Shh signaling pathway [30–33]. In order to clarify whether GSK-3*β* provided a bridge between Shh and PI3K/Akt, GSK-3*β* phosphorylation was investigated. It was found that phosphorylated GSK-3*β* significantly decreased when PI3K/Akt was blocked by PI3K inhibitor. Meanwhile, the blockage of Shh pathway could downregulate phosphorylated GSK-3*β* by reducing Gli1 expression to affect Akt phosphorylation. We thereby consider that an interaction between Shh and PI3K/Akt pathways through modulating GSK-3*β* expression may be involved in TMT-induced apoptosis in N2a cells. Specifically speaking, PI3K/Akt pathway could influence GSK-3*β* expression to regulate Shh signaling, whereas Shh signaling was able to regulate Gli1 and GSK-3*β* expression to mediate PI3K/Akt signaling.

LBP is widely used in the manufacture of health care products to improve immunity, neuronal protection, and antiaging [14, 18, 34]. In a variety of methods that were used to relieve nerve damage caused by TMT, LBP could be a good choice because of its advantages of being cheap and available. Our preliminary experiment showed that a concentration range of 50~500 *μ*g/mL LBP significantly improved cell viability, in which the concentration of LBP at 300 *μ*g/mL played best effect on TMT-induced cytotoxicity in N2a cells [14, 18, 34]. Many studies have shown that LBP could scavenge free radicals and reduce lipid peroxidation [35]. LBP not only exhibits a protective effect on A*β* caused neurotoxicity, but also inhibits the formation of traumatic neuroma [36]. In this study, LBP significantly reduced oxidation-promoting factors levels, such as ROS and MDA, and antioxidant SOD levels, upregulated antiapoptotic genes Bcl-2, downregulated proapoptotic genes Bax, lowered caspase-3 activity, and protected against neuronal apoptosis in TMT-treated N2a cells. In the aspect of mechanisms, the protective pathway PI3K/Akt signaling was activated after LBP treatment. The Shh signaling pathway downstream Gli1 gene expression also markedly increased compared with TMT treatment. The phosphorylated GSK-3*β* reduction by TMT was reversed by LBP addition. Thus, LBP could antagonize TMT-induced toxicity by exerting antioxidative effect and initiating Shh and PI3K/Akt signaling pathways in N2a cells. LBP can be proposed as a protective agent against TMT neurotoxicity in clinic.

## 5. Conclusion

**Figure 9:**
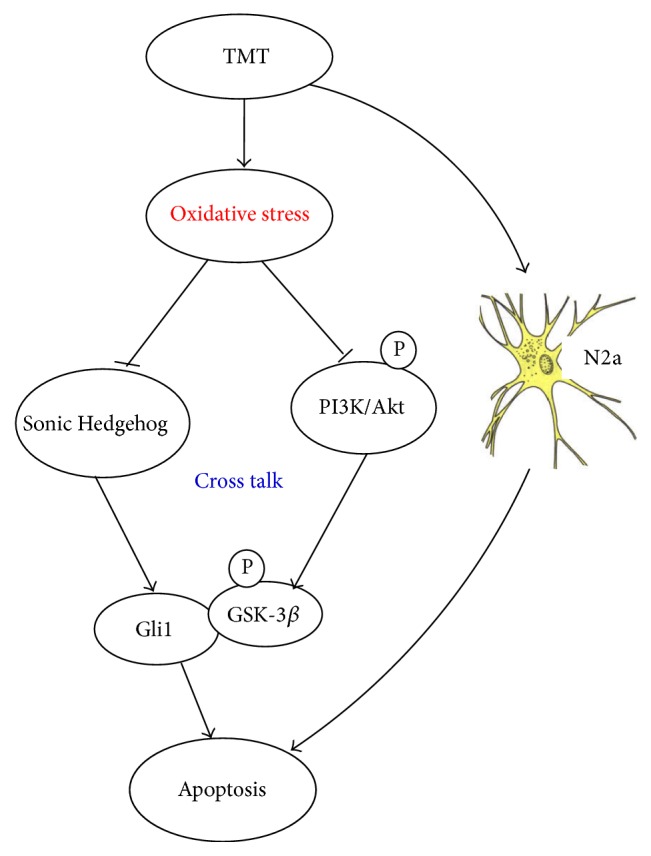
Mechanisms of TMT-induced apoptosis in N2a cells.

In brief, TMT treatment induced neuronal apoptosis by causing oxidative stress, regulating apoptotic genes, and inhibiting Shh and PI3K/Akt signaling pathways. An interaction between Shh and PI3K/Akt signaling pathways is confirmed. LBP played a protective role in TMT-induced neurotoxicity by decreasing oxidative stress and activating Shh and PI3K/Akt signaling pathway. These findings suggest that Shh and PI3K/Akt signaling pathways may be potential therapeutic targets in the molecule mechanisms of TMT-induced nerve damage (Figure 9).

## Abbreviations

TMT: Trimethyltin chloride
LBP: * Lycium barbarum* polysaccharides
Shh: Sonic Hedgehog
GSK-3*β*: Glycogen synthase kinase-3*β*
N2a: Neuro-2a
ROS: Reactive oxygen species
MDA: Malondialdehyde
SOD: Superoxide dismutase
PD: Parkinson's disease
AD: Alzheimer's disease
DMEM: Dulbecco's modified Eagle's medium
MTT: 3-(4,5-Dimethylthiazol-2-yl)-2,5-diphenyltetrazolium bromide
DCFH-DA: 2,7-Dichlorofluorescein diacetate
FBS: Fetal bovine serum
DMSO: Dimethyl sulfoxide
TBA: Thiobarbituric acid.
